# Supplementary material for: The effects of viewing a winter forest landscape with the ground and trees covered in snow on the psychological relaxation of young Finnish adults: A pilot study
Source: PLoS One. 2021 Jan 7;16(1):e0244799. doi: 10.1371/journal.pone.0244799 (PMC7790245; doi:10.1371/journal.pone.0244799)
Supplement: Attachment — Submitted filename: Review PLOS ONE 09.11.2020.docx [file pone.0244799.s001.docx]

**Reviewer #1:**

We thank the reviewer for these comments, we hope our responses to these comments are sufficient, and appreciate the reviewer's contribution to improving the quality of this work.

In the current version of the manuscript, Authors recognize several limitations of the research and they use a more cautious title. Nevertheless, there is a statements that should be dampened. The statement, "In other studies [56,57] the number of participants was 12-15, which was sufficient to draw meaningful conclusions" should be reworded in the following way: "In other studies [56,57] the number of participants was 12-15, which HAS BEEN CONSIDERED sufficient to draw PRELIMINARY conclusions." Previous underpowered studies are not a justification to carry out future studies underpowered.

Thanks to reviewer # 1 for this comment, of course we made some corrections.

Even though the manuscript report limitations, Authors recognize such limitations in the discussion section and provide some suggestions for future studies. Moreover, the topic is somewhat original and might stimulate future research. Thus, in my view, the manuscript could be accepted as a pilot study after this round of minor revision by which Authors dampen the statement above reported.

Thank you to the reviewer for appreciating this topic and our corrections.

**Reviewer #2:**

Part of my concerns have been adressed by authors. Unfortunately, there are important issues that continue to be present in the paper like uncorrect use of some terms and not clear statements that have to be corrected or clarified. In general, the study should be described with greater methodological rigor.

We thank the reviewer for these comments, we hope our responses to these comments are sufficient, and appreciate the reviewer's contribution to improving the quality of this work.

Examples are:
-in my opinion, the term "stimulation" used to describe experimental conditions should be substituted with "exposition"

Thank you for this comment, we've made some corrections.

- you define the sample as "healthy" but this statement is not supported with any test (line 128)

Thank you for this comment, we have removed this adjective.

- if groups are equal in composition and experimental conditions they received, why did you define two groups as experimental and control (e.g., line 138)? In statistical analyses section you use a within design. You should clarify this aspect

We thank the reviewer for this comment. Of course we meant group A and group B. It was a crossover study (control and experimental groups were exchanging, so this is the same as group A and group B). We have supplemented this in the text.

- In lines 73-74, bibliographic references are necessary. It is a crucial point of the paper because you operatively define the "psychological relaxation effect".

We thank the reviewer for this comment. We have added needed literature here.

- in both buildings and forest conditions subjects could stay stayed or sat. Given the importance of these two condition on "psychological relaxation effect", this aspect should to be controlled.

We thank the reviewer for this comment. Unfortunately, we have not controlled this effect, so we consider it a research limitation (if the reviewer kindly allows). We added an explanation:

“Another limitation of the research was the differentiation of the way of psychological relaxation - the subjects could rest while sitting or standing and look at the forest. However, this variable, ie sitting or standing, was not controlled in the experiment.”

\
-"sample size of 0.25" should be corrected with "effect size of 0.25" (line 143)

We thank the reviewer for this comment. Yes, we have corrected it.

-lines 136-138 are not clear

We thank the reviewer for this comment. We clarified it, it was about groups A and B (	which are interchangeably control and corossover study group).

- you should clarify if the climate features (e.g., temperature) are the same in "forest" and "building" conditions. Indeed, the snow was a prominent feature of forest condition, but you didn't report the state of snow in building condition.

We thank the reviewer for this comment. We have added the following text:

„The snow cover layer was similar in both experimental sites (in the forest and in front of the buildings). The climatic conditions were similar in both experimental sites, because there was no strong wind on that day, the differences were not felt by the respondents (oral report).”

and in limitations:

“Another limitation may be the fact that the climatic conditions of the environment were not accurately measured in each experimental site (in the forest and in front of buildings). Both of these limitations will be considered and eliminated in the next experiment.”

-lines 349-354 are not clear, you should explain better these concepts and support them with bibliografic references

Thank you to recent for this comment. We have added the following comment:

“This means that we believe that, under certain circumstances, snow can suppress the feeling of vigor as it blocks the stimulating effect of green in the forest and the view of trees. This stimulating effect has been proven in other studies”

-in line 371 what do you mean with "rough control"?

Thank you to recent for this comment. We've added the following text, and used this statement in quotation marks.

“(control in an experiment in the city, related to, inter alia, car traffic).”

-lines 480-481 should be clarified

Thank you to recent for this comment. We dropped that sentence - it was deleted.
