# Supplementary material for: The effects of viewing a winter forest landscape with the ground and trees covered in snow on the psychological relaxation of young Finnish adults: A pilot study
Source: PLoS One. 2021 Jan 7;16(1):e0244799. doi: 10.1371/journal.pone.0244799 (PMC7790245; doi:10.1371/journal.pone.0244799)
Supplement: Attachment — Submitted filename: Response to reviewers.docx [file pone.0244799.s002.docx]

**Response to reviewers:**

**Reviewer #1: (No Response)**

**Reply:**

We thank the reviewer for this comment.

**Reviewer #2:** Comments and concerns have been addressed. Limitations have been properly highlighted. I suggest to authors a further English check.

**Reply:**

We would like to thank the reviewer for this comment, we have made language corrections as a result of an additional language check two times by qualified proofreader and also proofreading services.

**Reviewer #3:**

- It has been already said that the experimental subjects were randomly assigned to the two groups (line 133), there is no need to repeat it in line 179

**Reply:**

We thank the reviewer for this comment, we have removed the repetitive fragment of the text.

- In lines 359-36: "the other strong hypothesis is that the snow covered environment might influence them not only by visual stimulation but also through a reduction in the concentration of volatiles in the air". This aspect is not directly measured, so it may be more appropriate to re-evaluate the term "strong hypothesis" used in the paper

**Reply:**

We thank the reviewer for this comment, we reevaluated the meaning of this sequence, removed the word "strong".
